# Extending the depth range in energy-dispersive X-ray stress analysis by simultaneous multi-detector data acquisition in equatorial scattering geometry

**DOI:** 10.1107/S1600576725007599

**Published:** 2025-09-24

**Authors:** Christoph Genzel, Daniel Apel, Mirko Boin, Manuela Klaus

**Affiliations:** a Department of Microstructure and Residual Stress Analysis Helmholtz-Zentrum Berlin für Materialien und Energie Germany; Montanuniversität Leoben, Austria

**Keywords:** X-ray stress analysis, energy-dispersive diffraction, two-detector setups, horizontal scattering geometry, stress gradients, polycrystalline materials

## Abstract

A scattering geometry for depth-resolved energy-dispersive X-ray stress analysis on polycrystalline materials is introduced. Via simultaneous data acquisition during a sin^2^ ψ measurement using two detectors arranged in the horizontal diffraction plane, it aims to extend the accessible information depth to the free surface as well as deeper material zones. While data acquisition with the first detector takes place in a symmetrical configuration with regard to the incident and exit angles, α_i_ and α_e_, respectively, the second detector runs in an asymmetrical mode, defined by α_i_ < α_e_. Therefore, the scattering vectors assigned to the two diffraction geometries run in different tilt planes during a χ scan of the sample performed in the Eulerian cradle. Treatment of the data recorded in the asymmetric diffraction mode requires modifications of the fundamental equation of X-ray stress analysis, which are discussed using the example of measurements performed on a unidirectionally ground ferritic steel sample.

## Abstract 2

Energy-dispersive diffraction using a two-detector setup in horizontal scattering geometry is applied for depth-resolved residual stress analysis. Data evaluation based on a modified fundamental equation is discussed via measurements performed on a unidirectionally ground ferritic steel sample.

## 1. Introduction

Compared with angle-dispersive (AD) diffraction, the method of energy-dispersive (ED) diffraction introduced by Giessen & Gordon (1968 ▸) and Buras *et al.* (1968 ▸) provides complete diffraction patterns in fixed but freely selectable scattering directions 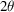. Bragg’s law in its ED form reads 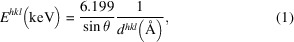
where 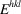 and 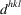 denote the photon energy of the diffraction line *hkl* and the corresponding lattice spacing, respectively. From equation (1) it follows that a smaller (larger) Bragg angle θ results in a ‘stretching’ (‘compression’) of the diffraction spectrum towards larger (smaller) energies. These two features, the freely selectable scattering direction and the tunable energy range, enable data acquisition using two or even (many) more detectors for simultaneous mapping of different orientations with respect to the sample reference system. Examples of experimental stations at large-scale facilities equipped with a series of detectors that are arranged around the primary beam to cover a wide 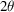 range are the synchrotron X-ray beamline I12 at the Diamond Light Source (Drakopoulos *et al.*, 2015 ▸) and the neutron diffractometer GEM at ISIS (Hannon, 2005 ▸). ED (time of flight) neutron strain scanning experiments are usually equipped with two detectors that are aligned perpendicular to the primary beam in opposite directions to allow simultaneous data acquisition in different directions (Bourke *et al.*, 2002 ▸; Santisteban *et al.*, 2006 ▸).

In ED X-ray stress analysis (ED-XSA), multi-detector setups can be used in various configurations to detect the lattice strain simultaneously in different measuring directions. Defining a small gauge volume by narrow slits in the primary and diffracted beam, a horizontal detector array behind the secondary slit system enables real-space residual stress depth profiling by through-surface strain scanning in reflection geometry (Denks, 2008 ▸; Genzel *et al.*, 2013 ▸; Meixner *et al.*, 2015*a* ▸,*b* ▸). A similar setup was used by Genzel *et al.* (2011 ▸) to study the stress evolution during thin-film growth.

A natural depth resolution in ED diffraction experiments performed in reflection geometry is given by the different photon energies 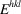 of the reflections *hkl* in the diffraction pattern. The 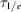 information depth, where 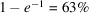 of the total intensity diffracted by a thick sample originates from, is given by 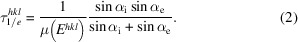
Here μ is the linear absorption coefficient depending on the photon energy, and 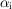 and 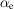 denote the angles that the sample surface forms with the incoming and the diffracted X-ray beam, respectively. This feature of ED diffraction was used by Ruppersberg (1997 ▸) for depth-resolved ED-XSA based on his universal plot method (Ruppersberg *et al.*, 1989 ▸, 1991 ▸).

ED-XSA utilizing the 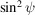 method (Macherauch & Müller, 1961 ▸) also enables the evaluation of near-surface residual stress depth profiles. The idea of combining the information from 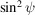 analyses using different wavelengths and reflections *hkl* to generate discrete residual stress depth profiles was introduced by Eigenmann *et al.* (1990 ▸). The multi-wavelength method is based on AD diffraction experiments, which require a change of X-ray tubes to vary the characteristic wavelength and, therefore, the photon energy.

The transfer of the multi-wavelength approach to the ED case, introduced as the ‘modified multi-wavelength’ or MMW method (Genzel *et al.*, 2004 ▸), offers the advantage that different information depths are already represented in each diffraction spectrum by the discrete reflections *hkl* with different photon energies 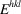. In this case, linear regression is applied to the 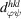
–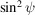 distributions (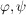 – azimuth and inclination angle of the scattering vector in the sample reference system, respectively) of any evaluable reflection *hkl* in the diffracted spectrum. Plotting the stress values obtained this way versus the maximum information depth, which corresponds to 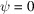, yields discrete profiles 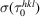 in the Laplace space that can be transformed back into the real space to obtain 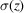 (Klaus & Genzel, 2019 ▸).

The present work adopts the MMW approach and develops it further. The corresponding experiments were carried out on an eight-circle diffractometer system (LEDDI – laboratory ED diffraction) equipped with two ED detectors, D1 and D2, which can be moved independently in the horizontal and vertical planes. Apel *et al.* (2018*a* ▸,*b* ▸) showed that appropriate combinations of a pure vertical (D1) and an inclined (D2) scattering geometry provide simultaneous depth profiles for the in-plane normal and out-of-plane shear stress components by a single χ scan of the sample in the Eulerian cradle between 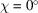 and 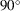 (mode 1) and 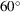 (mode 2), respectively. For both modes, the scattering vectors assigned to the two scattering geometries lie in the same plane during the χ tilt, which ensures that both the positive and the negative ψ branch are covered during the scan. Consequently, the number of nodes 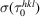 in the depth profiles is restricted to the intersection of identical reflections *hkl* that are registered in the two detectors.

**Figure 1:**
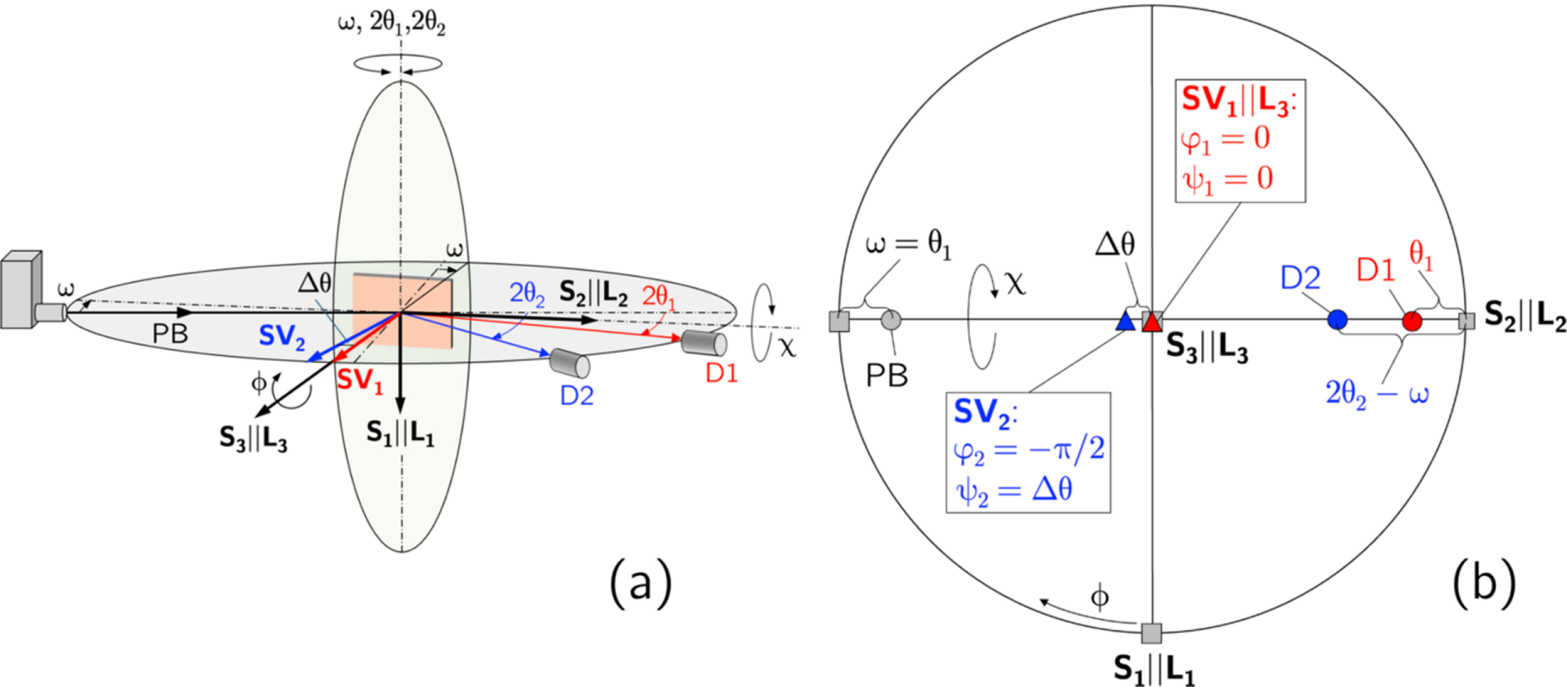
(*a*) Schematic view and (*b*) stereographic representation of the horizontal two-detector configuration. Elements belonging to detector D1 (D2) are represented in red (blue). In the initial setup shown here, the sample coordinate system 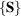 coincides with the laboratory system 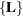. The diffraction plane spanned by the primary beam (PB) and by the two beams diffracted into detectors D1 and D2 contains the two scattering vectors 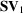 and 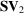. Symmetric/asymmetric diffraction conditions are assumed for detector D1/D2, which are characterized by 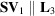 and 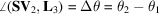, respectively. For further details, see the main text.

The approach introduced in the present paper aims to increase the number of nodes in the Laplace stress depth profiles, 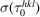, to improve the conditions for transforming them back into real-space profiles, 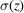. For this purpose, the X-ray source and the two detectors form a common scattering plane (Fig. 1 ▸). According to equation (1), the two detectors provide diffraction spectra which, in the case of 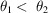 as applied in the present paper, are ‘stretched’ towards higher energies (D1) and ‘compressed’ towards lower energies (D2). A single 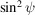 measurement via a χ scan therefore provides two Laplace stress depth profiles, 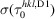 and 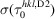, which are interlocked with each other and merge to form an overall sum profile.

However, the operation of two detectors in the same scattering plane implies that symmetric diffraction conditions can only be realized for one of them. The assumption of a symmetrical diffraction geometry for detector D1 leads to an asymmetric geometry for the second detector D2. In this case, a sample tilt around an axis fixed by the intersection of the sample surface and the scattering plane (see Fig. 1 ▸) means a 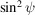 measurement in the Ψ mode of XSA (Hauk & Macherauch, 1984 ▸) for detector D1, and a measurement in a ‘mixed mode’ for detector D2. The latter mode is characterized by the feature that the scattering vector 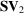 is no longer perpendicular to the tilt axis. Using a very small incidence angle, this ‘asymmetric Ψ mode’ had been introduced by Dümmer *et al.* (1999 ▸) to restrict the information depth for residual stress analysis performed in the AD diffraction mode to the near-surface region of thin films. The same approach was exploited by Kumar *et al.* (2006*b* ▸,*a* ▸) for residual stress analysis at fixed/predefined information depths. A slightly different method called ‘asymmetric 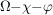 mode’ that also aims at the analysis of residual stresses by keeping the information depth fixed was suggested by Erbacher *et al.* (2008 ▸).

**Figure 2:**
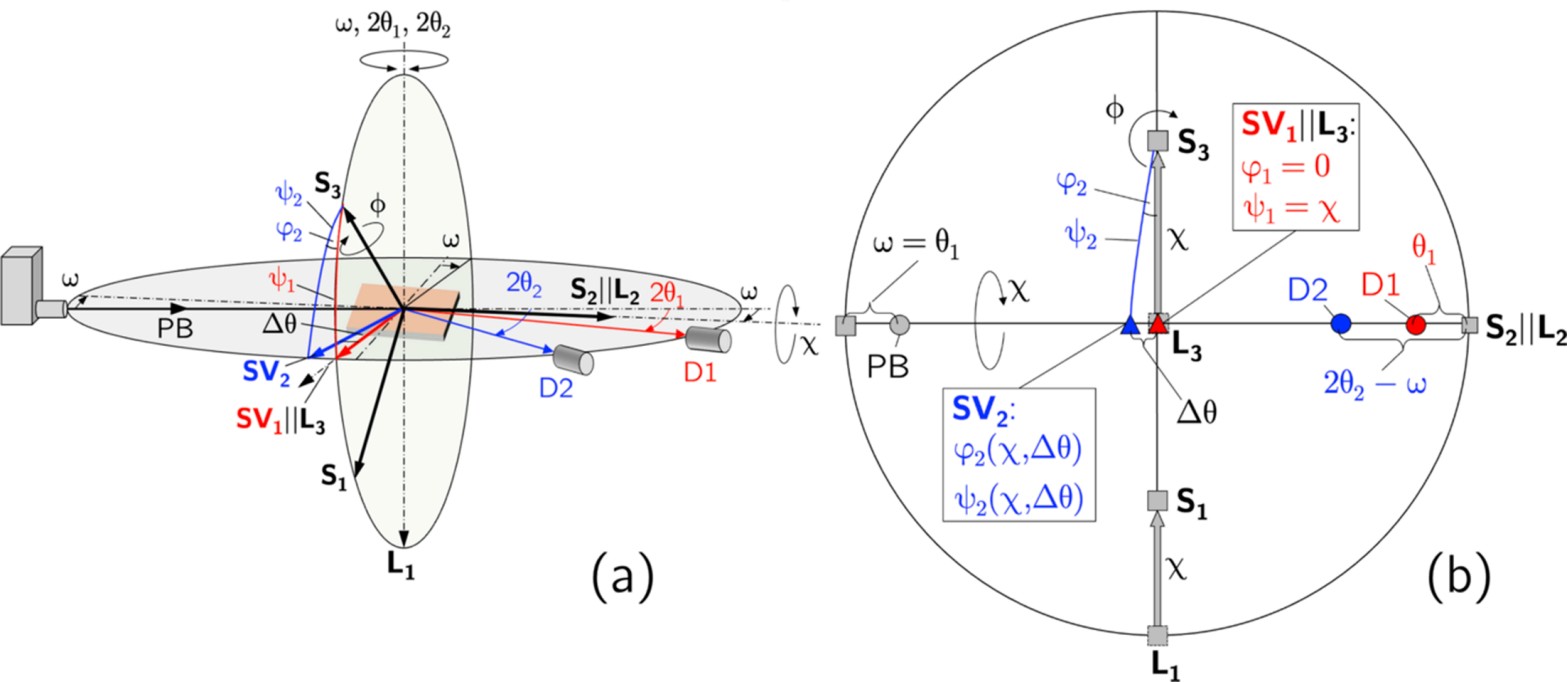
The horizontal two-detector configuration for an inclined sample orientation, realized by a χ tilt around an axis defined by the intersection line between the sample surface and the diffraction plane. (*a*) Schematic view and (*b*) stereographic representation of the setup. For the descriptions of the axes and angles, see Fig. 1 ▸.

What all the asymmetric XSA methods have in common is that the stepwise sample tilt requires a successive readjustment of the angle φ to fix the azimuth orientation of the scattering vector in the case of a non-rotationally symmetric in-plane residual stress state. In the present study we combine the symmetric and the asymmetric Ψ mode in one χ scan for simultaneous data acquisition (see Fig. 2 ▸). Therefore, as an azimuthal sample rotation during the χ scan is excluded, the data set obtained for detector D2, which is operated in the asymmetric mode, needs to be corrected. This results in a modification of the fundamental equation of XSA for D2, which, as shown in the following by an experimental example, takes into account the influence of the in- and out-of-plane stress components acting perpendicular to the azimuth direction covered in the χ scan.

## 2. The horizontal two-detector setup

### 2.1. Angles in the laboratory and sample coordinate systems

The horizontal two-detector setup used for the residual stress analysis leads to different relationships between the angle sets defined in the laboratory (diffractometer) system 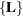 and sample coordinate system 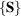 for the two detectors D1 and D2. According to Figs. 1 ▸ and 2 ▸, which show the diffractometer setup for the initial and an inclined sample orientation, respectively, we define the following:

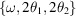
: goniometer angles in the laboratory system for aligning the sample (ω) and the detectors D1 (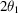) and D2 (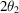) in the horizontal diffraction plane.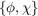: goniometer angles to adjust the azimuth (ϕ) and polar (χ) orientation of the sample with respect to the sample reference system.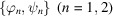: azimuth and polar angles, respectively, which define the orientation of the scattering vectors 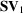 and 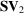 within the sample coordinate system for the detectors D1 and D2.

The following relationships hold between the angles in the laboratory (diffractometer) and in the sample coordinate systems: 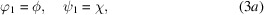

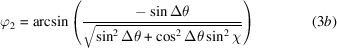
and 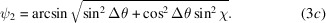
These relationships are used in the following to express the fundamental equation of XSA for both detectors uniformly in terms of the diffractometer angles.

### 2.2. The fundamental equation of XSA for detectors D1 and D2

For detector D1, a χ scan is identical to a 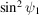 measurement in the symmetric Ψ mode. Thus, according to equation (3*a*[Disp-formula fd3]), the angle set 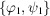 can be replaced by 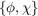 in the fundamental equation, which then reads (Stickforth, 1966 ▸) 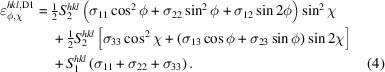
Here, 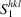 and 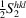 are the diffraction elastic constants (DEC), which can be calculated from the single-crystal elastic constants by assuming different interaction models. These include homogeneous strain (Voigt, 1910 ▸) or stress (Reuss, 1929 ▸) in all crystallites independent of their orientation, the arithmetic average of these two approaches (Neerfeld, 1942 ▸; Hill, 1952 ▸), or the more sophisticated model introduced by Eshelby (1957 ▸) and Kröner (1958 ▸), assuming elastic polarizability.

Concerning detector D2, 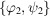 depend on 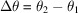 and χ according to equations (3*b*[Disp-formula fd4]) and (3*c*[Disp-formula fd5]). As a result, the explicit ϕ dependency of the individual stress components in the fundamental equation gets lost. Instead, the whole equation must now be considered separately for the different azimuths. This leads to the following relations: 
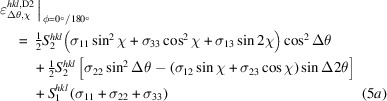
and 
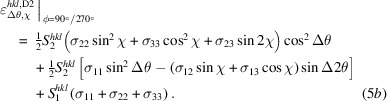
The above equations can be interpreted as follows. The first terms in round brackets contain those stress components that generate the slope and most of the splitting of the lattice strain distributions during a sample tilt χ into an azimuth direction ϕ. The stress components in the terms in square brackets, on the other hand, act transverse to the respective azimuthal tilt directions ϕ. They do not contribute to the slope (no 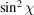 dependency), but due to the dependency on 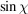 and 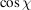 they contribute a certain amount (depending on 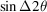) to χ splitting. A general discussion of the impact of all stress components on the lattice strain distributions would be beyond the scope of this paper. In the following, we will therefore limit ourselves to a realistic scenario, both with regard to the stress state itself [see equation (7) in Section 2.3] and with regard to the definition of the azimuthal measurement directions (either parallel or transverse to the grinding direction).

**Figure 3:**
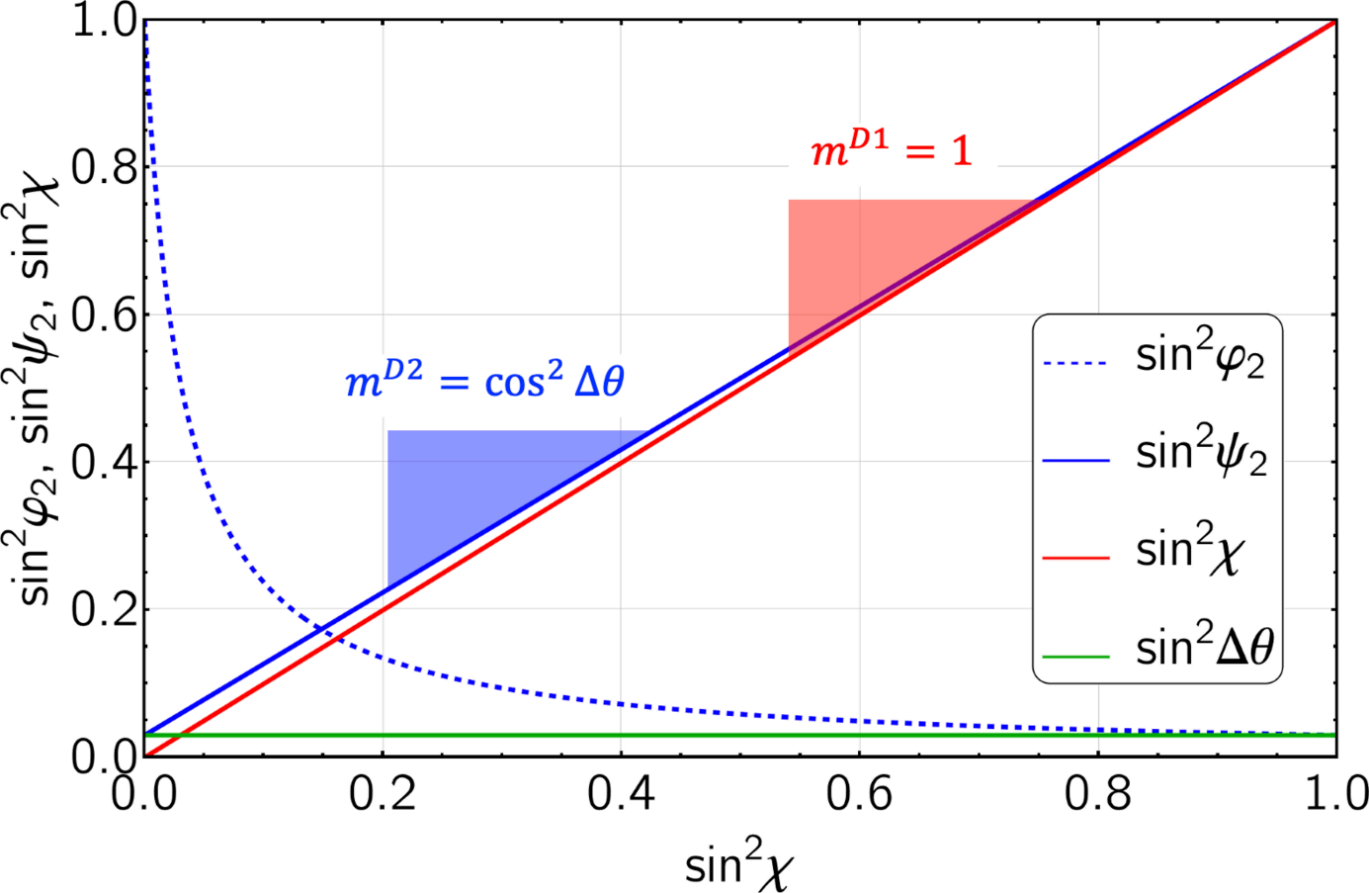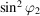 and 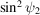 as functions of 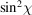 [*cf*. equations (3*b*[Disp-formula fd4]), (3*c*[Disp-formula fd5]) and (6)]. The difference 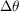 between the Bragg angles 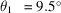 and 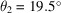 is 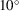, resulting in a correction factor 
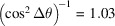
.

Due to the inclination of 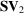 against the surface normal 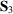 in the initial sample setup 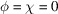, 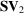 undergoes a reorientation in the sample reference system both in azimuth (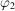) and in polar direction (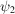) during a χ scan. From equation (3*c*[Disp-formula fd5]) we have 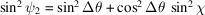 and thus 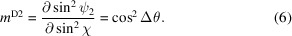
This means that the slope 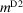 of the regression line fitted to the 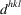
–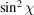 data obtained for detector D2 has to be corrected by a factor 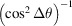 that depends on the difference of the Bragg angles used to record the diffraction spectra with the two detectors D1 and D2. To estimate an upper limit for the correction, consider that 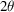 used in ED diffraction is typically between about 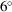 (diffraction spectrum ‘stretched’ towards high energies) and 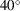 (diffraction spectrum ‘compressed’ towards low energies). Therefore, assuming that the maximum difference in Bragg angles is 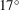, the correction factor would not exceed a value of 1.09, or in other words, the stress in the plane would be underestimated by 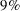 if the D2 data were evaluated without correction. Fig. 3 ▸ shows the situation for the scattering geometry used in the present paper.

Furthermore, the diagram reveals that the scattering vector 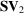 performs an azimuthal rotation around the 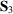 axis of the sample reference system by nearly 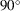. The consequences for residual stress analysis in the case of a non-rotationally symmetric in-plane residual stress state (*i.e.*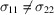) and in the presence of uniaxial shear stress 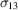 are discussed in the following section.

### 2.3. Normal and shear stress analysis with the horizontal two-detector setup

We consider a residual stress state that is frequently observed as a result of a uniaxial grinding process in the near-surface sample region:^1^
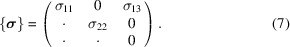
The following investigations aim to find out how the individual stress components are ‘perceived’ by the two detectors D1 and D2 during χ scans performed in different azimuth directions ϕ. As stress analysis by means of the 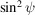 method is based on linear regression, we restrict our considerations on the non-constant terms of the fundamental equation, which contain 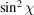 and 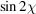.

**Figure 4:**
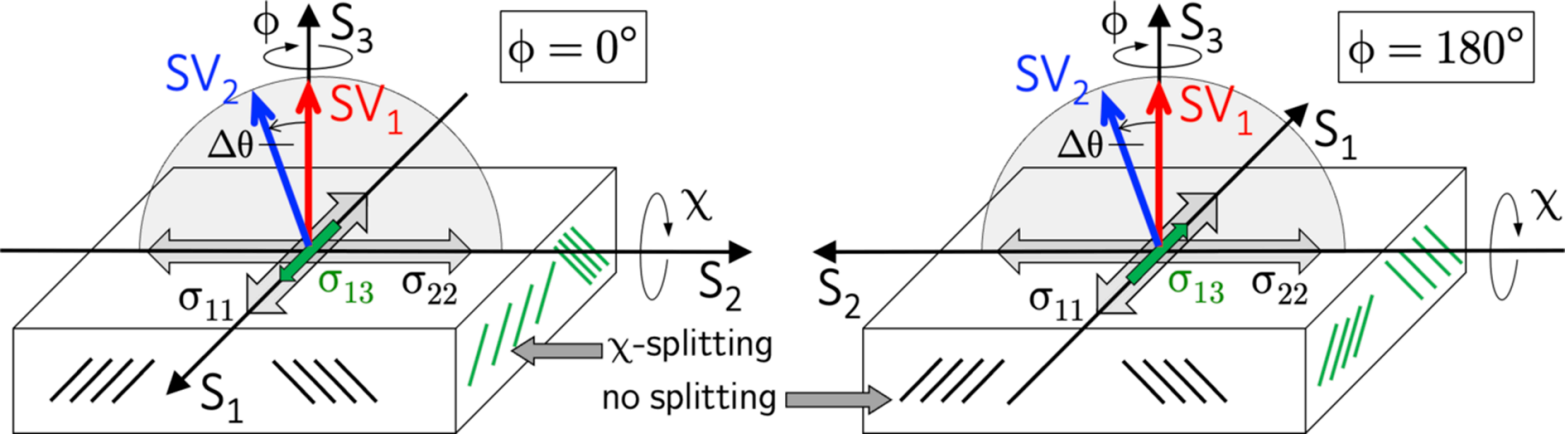
Schematic view of stress analysis along the grinding direction on a sample featuring a residual stress state according to equation (7). For visualization reasons, the diffraction plane, which contains the two scattering vectors 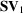 and 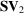, is shown tilted by 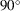 into the paper plane.

For χ scans carried out in the azimuths 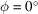 and 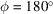, *i.e.* parallel and antiparallel to the grinding direction, the shear stress 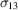 will cause χ splitting, which can be exploited to separate this component in the evaluation from the in-plane normal component 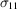 (Hauk, 1997 ▸). From equations (4), (5*a*[Disp-formula fd7]) and (5*b*[Disp-formula fd8]) we obtain 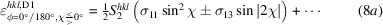
and 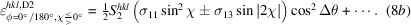
Fig. 4 ▸ illustrates that both detectors sense the elliptical splitting caused by the shear stress 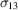. For detector D2, however, the observed effect is smaller by the amount 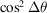, which is due to the inclination of the scattering vector 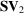 perpendicular to the measurement direction. This also applies to the mean slope of the lattice strain distributions caused by the in-plane component 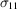 (*cf*. Fig. 3 ▸). Furthermore, equations (8*a*[Disp-formula fd11]) and (8*b*[Disp-formula fd12]) reveal that the detection of the two different χ branches, which is the precondition for the separation of 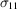 and 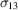, is possible in two ways. Using a 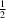-circle cradle both the positive and the negative χ branch are accessible for 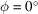 or 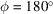 from one scan in the range 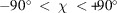. If only a 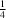-circle cradle is available, the χ splitting can be observed by two χ scans in the range 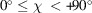 in the azimuths 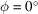 and 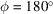 (Faninger & Walburger, 1976 ▸).

**Figure 5:**
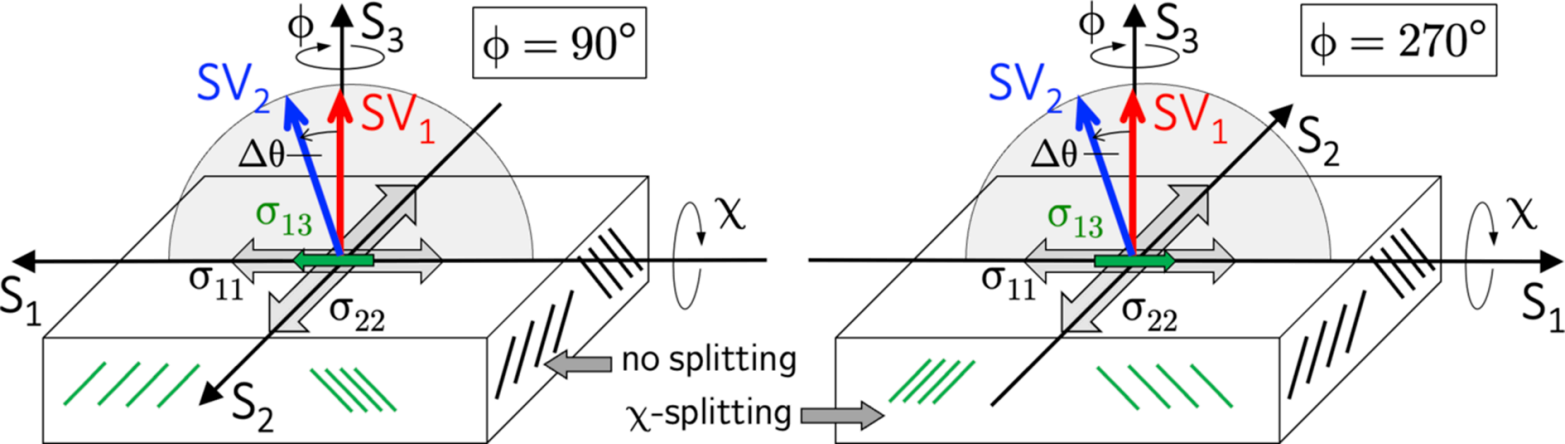
Schematic view of stress analysis perpendicular to the grinding direction (*cf*. also Fig. 4 ▸). See the main text for details.

For χ scans in the transverse direction performed in the azimuths 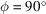 and 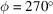, the following relationships apply to the lattice strains recorded in the two detectors: 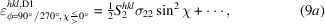

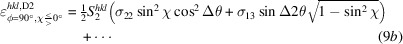
and 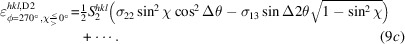
The meaning of the equations valid for the transverse direction can be explained by considering Fig. 5 ▸. Detector D1 does not ‘see’ the shear stress component 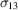 because the scattering vector 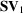 is perpendicular to the grinding direction during the χ scan. However, detector D2 will detect 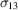, at least partially, as the scattering vector 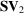 is inclined against the grinding direction by an amount 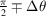, depending on which azimuth (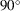 or 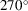) is chosen for the χ scan in the range 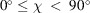. Equations (9*b*[Disp-formula fd14]) and (9*c*[Disp-formula fd15]) reveal an important consequence concerning the scanning mode applicable to detect the two χ branches with detector D2. In contrast to stress analysis along the grinding direction described by the equation (8*b*[Disp-formula fd12]), the term containing 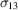 does not change its sign if χ scans in the range 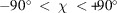 are done for only one azimuth. In these cases Fig. 5 ▸ shows that the lattice planes, which are stretched and compressed by the impact of the shear stress component, are always on the same side as the scattering vector 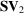 for one and the same azimuth angle ϕ. Therefore, the separation of 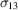 from the transverse in-plane normal stress component 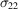 is only possible if χ scans in the range 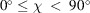 are performed in the two opposite azimuths 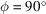 and 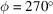. In other words, χ scans in the range 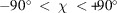 for the same azimuth (*i.e.*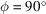 or 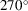) would lead to incorrect results for the stress component 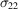.

## 3. Experimental

### 3.1. Sample material

**Figure 6:**
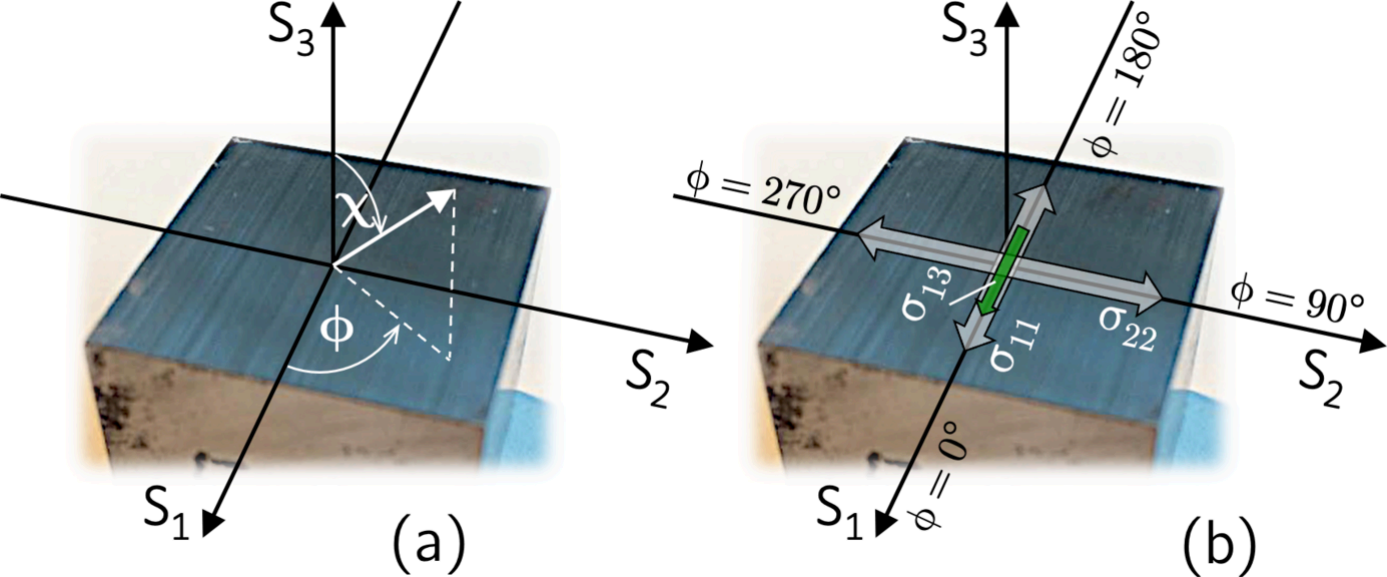
The analyzed specimen. (*a*) Alignment of the sample reference system 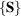 with respect to the edges of the specimen. (*b*) Orientation of the components of the stress tensor 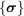 in relation to the grinding direction parallel to 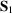.

To verify the approach proposed in this paper, measurements using the two-detector setup introduced in Section 2 were performed on a ferritic steel alloy C100 featuring a square base of 25 mm edge length and 20 mm height (Fig. 6 ▸). Details of the chemical composition and the heat treatment to generate a homogeneous fine-lamellar microstructure may be found in the work of Meixner *et al.* (2018 ▸). To generate a well-defined near-surface residual stress state, the specimen was uniaxially ground in 40 steps, generating 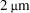 abrasion each. The surface treatment was performed in counter rotation mode with cubic boron nitride as abrasive.

### 3.2. X-ray diffraction and data evaluation

For the measurements, a tungsten X-ray tube with long fine focus was used. The operation conditions were 60 keV/45 mA. The primary X-ray beam optics consisted of a collimator that shaped the beam cross section to a diameter of 0.8 mm. Soller slits in front of the two KETEK Si-drift detectors were used to restrict the equatorial divergence of the diffracted beams to 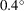. Then, χ scans in the range 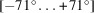 with a step width of 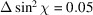 were carried out in the azimuths 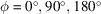 and 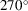. The counting time for simultaneous data acquisition of the diffraction spectra at 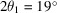 (detector D1) and 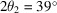 (detector D2) was 3600 s. The intensity distributions of all reflections in the ED diffraction spectra observed during the χ scans in the different azimuths showed a continuous decrease without local maxima and thus revealed the presence of a random crystallographic texture.

**Figure 7:**
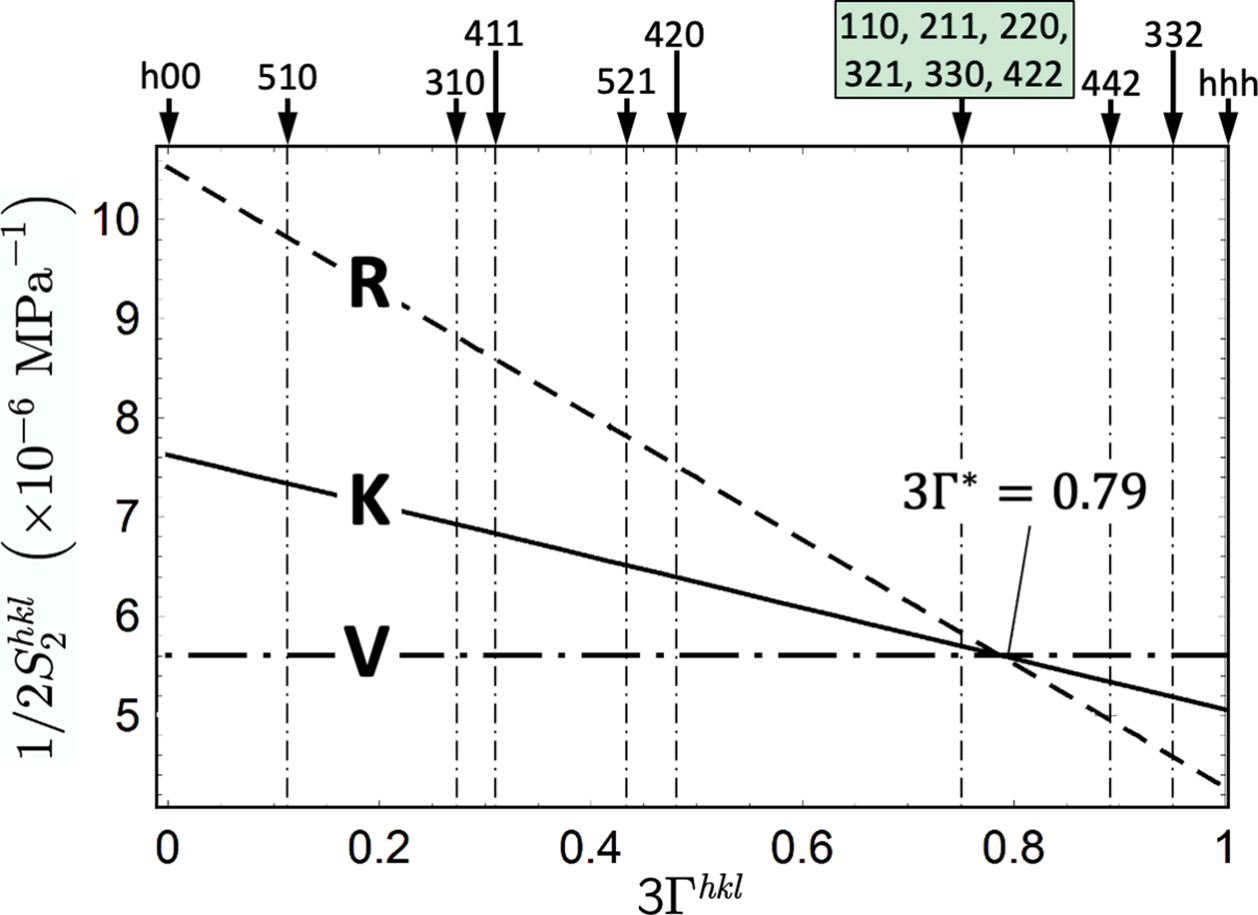
Diffraction elastic constant 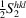 for ferritic steel, calculated for the grain-interaction models of Reuss (R), Voigt (V) and Eshelby–Kröner (K), and plotted versus the orientation factor 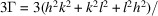

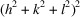
. The framed reflections *hkl* lie in the vicinity of the model-independent orientation 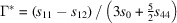 with 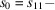

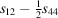
.

After absorption and background correction, the individual diffraction lines in the spectra were fitted using pseudo-Voigt functions (Apel *et al.*, 2020 ▸). The DEC required for stress evaluation were calculated from the single-crystal elastic constants 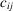 and moduli 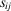 for ferritic steel taken from Landolt-Börnstein (1984*a* ▸,*b* ▸). Fig. 7 ▸ reveals that the 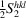 values for reflections *hkl* with an orientation factor 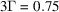 are close to the model-independent orientation 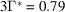 for ferritic steel, where the DEC for materials with cubic symmetry coincide for the different grain-interaction models. These reflections are of particular importance for depth-resolved stress analysis, as the stress values determined with them are independent of the assumptions regarding the underlying model.

**Figure 8:**
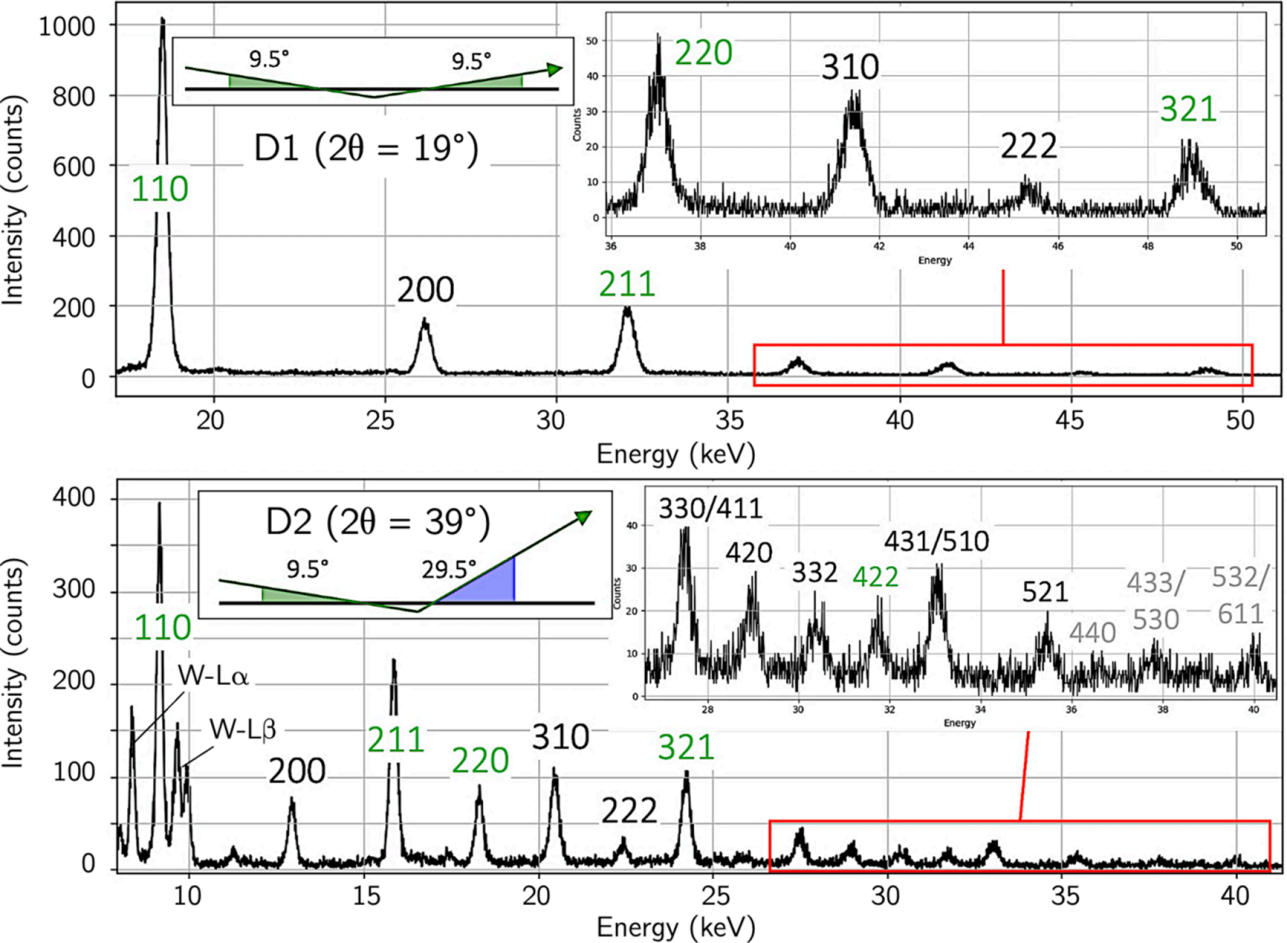
Diffraction patterns simultaneously recorded in the two detectors at 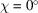. The diffraction conditions are symmetrical for detector D1 and asymmetrical (grazing incidence) for detector D2. The reflections marked in green feature the orientation factor 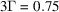 close to the model-independent orientation for ferritic steel (*cf*. Fig. 7 ▸). The reflections marked in gray are weak and were not considered in stress analysis.

Examples of diffraction patterns obtained with the two detectors are depicted in Fig. 8 ▸. According to equation (1), the ED spectra are stretched/compressed towards larger/smaller energies for detector D1/D2. Analyzable diffraction lines with sufficient intensity are available up to photon energies of about 50 keV (D1) or 25 keV (D2). One criterion for the choice of the 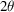 angle for detector D2 was to position the diffraction line 110 closest to the surface in respect of its information depth on the energy scale between the 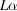 and 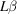 fluorescence lines of tungsten (see the lower diagram in Fig. 8 ▸).

**Figure 9:**
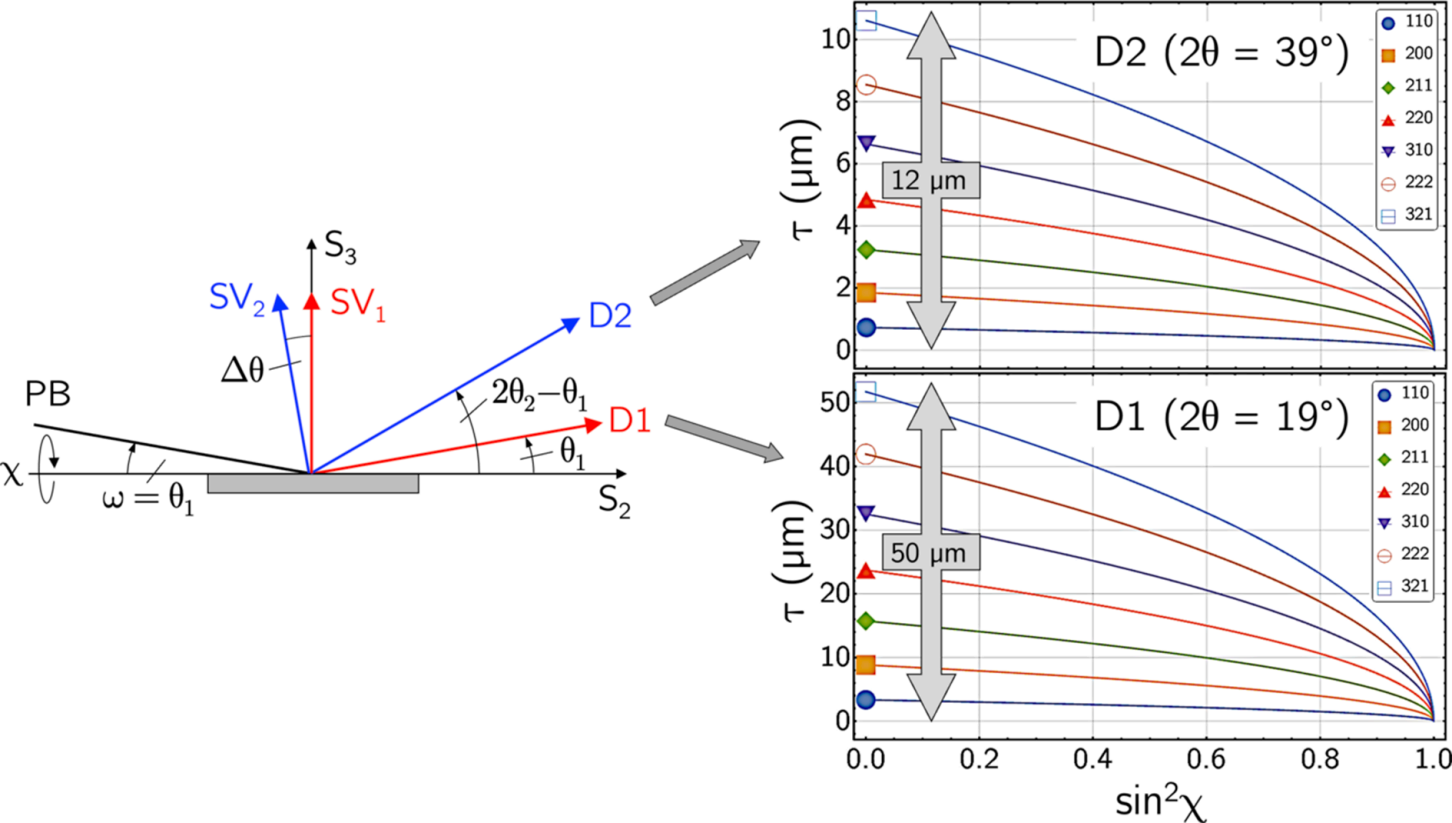
Information depths covered by detectors D1 and D2 according to equations (10*a*[Disp-formula fd16]) and (10*b*[Disp-formula fd17]), respectively.

The different energy ranges covered by the two detectors are reflected in the depth ranges captured in the near-surface materials region. From equation (2) one obtains 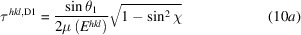
and 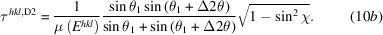
From Fig. 9 ▸, considering the same reflections *hkl* for both detectors leads to an approximately four times larger information depth for detector D1. The sets of information obtained with the two detectors therefore complement each other. While D2 is sensitive in the area close to the surface, where particularly steep residual stress gradients are often observed, D1 detects deeper material zones in which the residual stresses decrease or even change sign.

## 4. Results

### 4.1. Strain analysis

**Figure 10:**
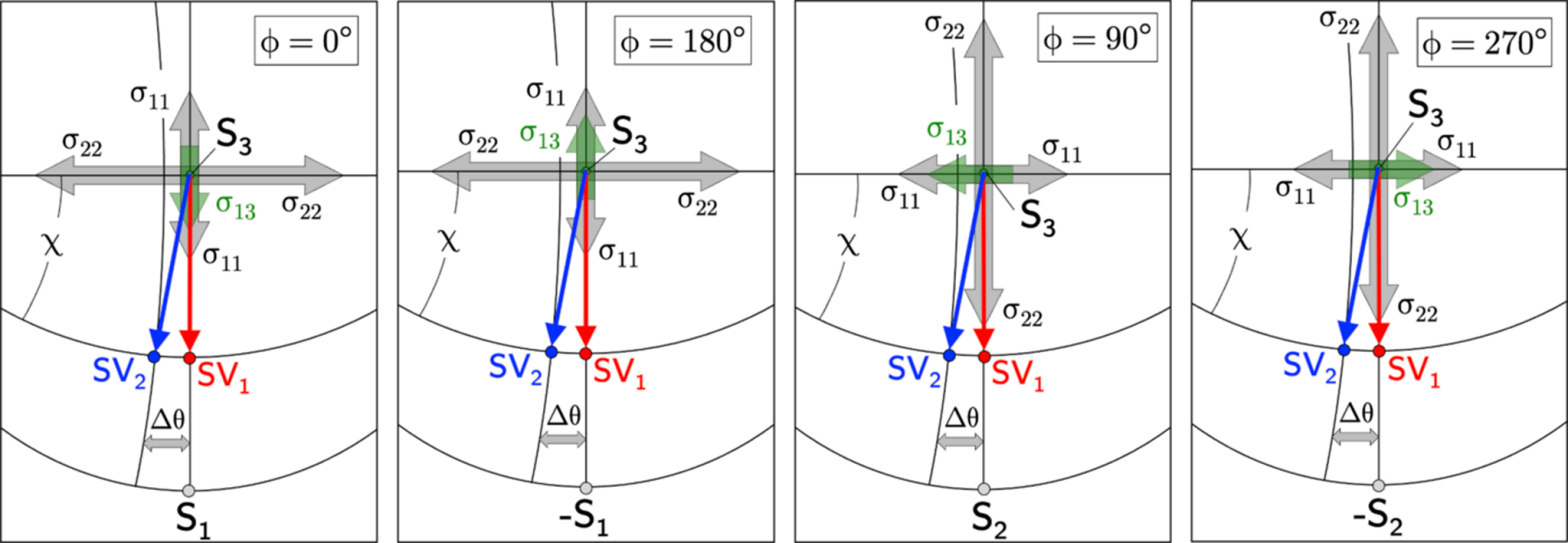
Stereographic projections showing the orientation of the two scattering vectors 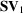 and 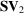 during the χ scans covering 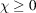 in the different azimuths with respect to the residual stress components that occur in the near-surface zone.

Fig. 10 ▸ demonstrates that the two detectors D1 and D2 will perceive the near-surface residual stress state differently during the χ scans performed in the four azimuths. The scattering vector 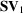 only tilts in planes that contain stress components or are perpendicular to them. Consequently, full (no) χ splitting will be observed with D1 in the azimuths 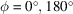 (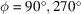). In contrast, the tilt plane of 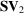 undergoes a continuous rotation during a χ scan (*cf*. Fig. 3 ▸). Therefore, the 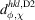
–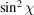 distributions recorded with detector D2 feature slopes that are, according to equations (8*b*[Disp-formula fd12]), (9*b*[Disp-formula fd14]) and (9*c*[Disp-formula fd15]), a factor of 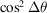 smaller than those distributions measured with D1. An interesting aspect for the measurements in the transverse direction has already been mentioned in Section 2.3: in the azimuths 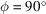 and 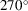, detector D2 will register the influence of the shear stress component 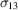 partially and differently, which leads to a certain χ splitting of the two branches.

**Figure 11:**
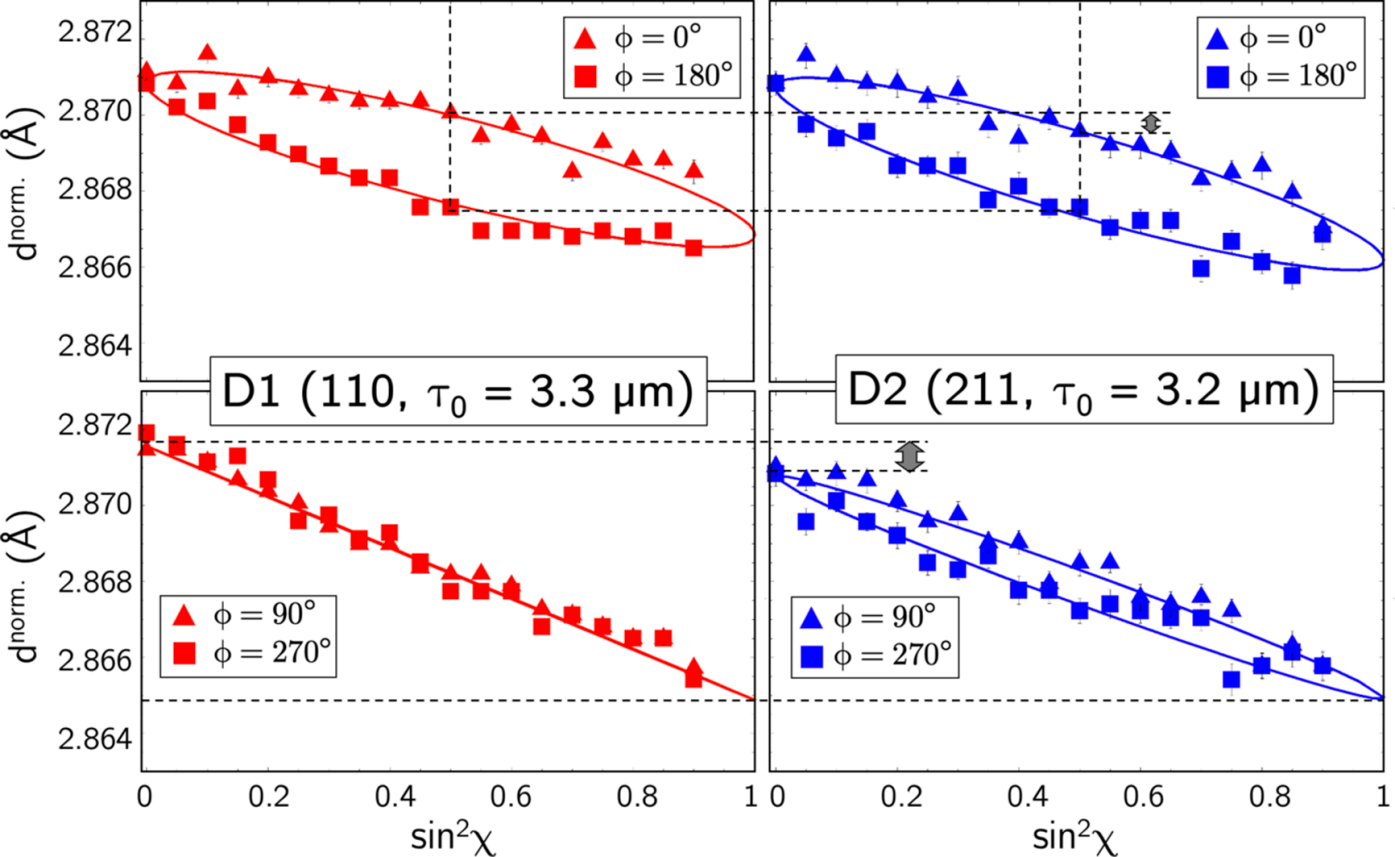
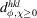
–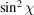 distributions for selected reflections *hkl* obtained with the horizontal two-detector setup. The gray double arrows mark the reduced χ splitting and slope of the D2 data.

Examples of the 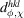
–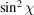 distributions determined with the two detectors are displayed in Fig. 11 ▸. For the purpose of comparability, the reflections were selected according to the following criteria: (1) the information should originate from the same (average) information depth to ensure that the same residual stress state is present, and (2) the DEC 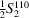 and 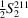 for the two reflections under consideration should be identical and independent of the grain-interaction model (see Fig. 7 ▸) to ensure that both the slope and the splitting of the distributions observed by the two detectors can be compared. The diagrams confirm the predictions made above on the basis of the stereographic projections shown in Fig. 10 ▸ with regard to the different detection of the residual stress state by the two detectors. This applies in particular to the measurements in the transverse direction, which do not reveal any χ splitting for detector D1, but do for detector D2. Furthermore, the impact of the factor 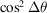 (which in the present case is 0.97) on the data obtained with detector D2 [*cf*. equations (8*b*[Disp-formula fd12]), (9*b*[Disp-formula fd14]) and (9*c*[Disp-formula fd15])] can be seen, which reduces both the χ splitting and the average slope of the 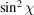 distributions.

**Figure 12:**
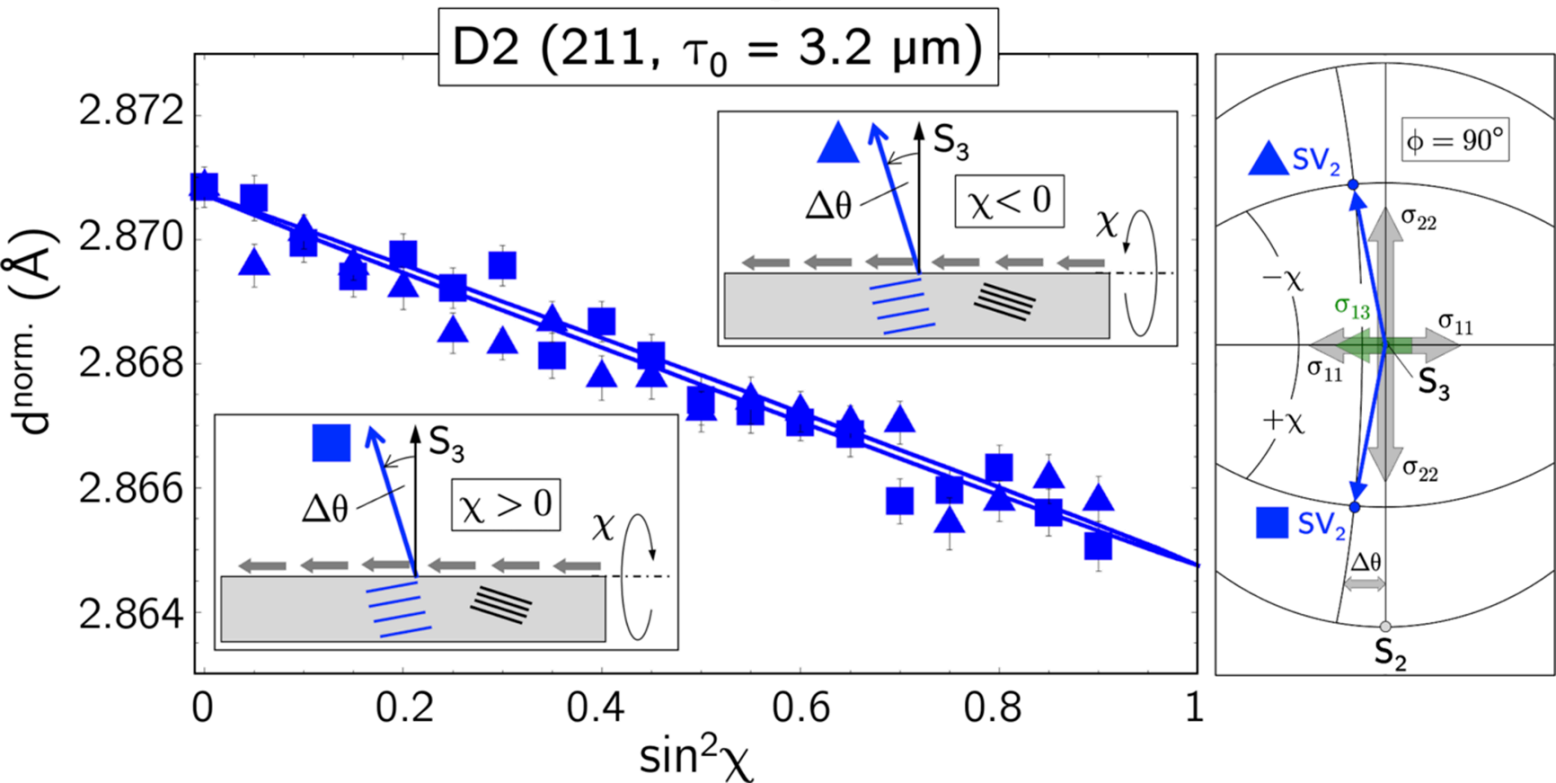
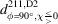
–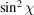 distribution obtained by measuring both the positive and the negative χ branch. See the main text for details.

Fig. 12 ▸ illustrates the situation where measurements are carried out in the transverse direction for the same azimuth (here, 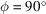) in both the positive and negative χ directions. The insets in the diagram and the stereographic projection on the right reveal that, although the entire χ range 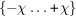 is covered, only one 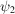 branch (here, the ‘stretched’ side, where the grinding process generated larger lattice spacings) is recognized. In this case, according to equation (9*b*[Disp-formula fd14]), the regression line fitted to the 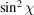 distribution would be influenced by the shear stress component 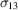 and therefore lead to an incorrect value for the stress component 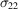.

### 4.2. Stress analysis

Residual stress evaluation on the basis of the 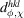
–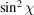 distributions received for the various reflections *hkl* with both detectors D1 and D2 was carried out in two steps. In the first step, linear regression was applied after combining the data measured in the different azimuths to separate the in-plane normal from the out-of-plane shear stress components. The error limits for the individual stress values are calculated according to the rules of error propagation. In the least-squares fit, each individual data point in the 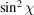 distribution is weighted according to its uncertainty. The residual stress values obtained for detector D2 were corrected by the factor 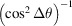 according to equation (6) (see also Fig. 3 ▸). Plotting the residual stresses versus the maximum information depths 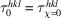 [*cf*. equations (10*a*[Disp-formula fd16]) and (10*b*[Disp-formula fd17])] results in discrete depth profiles 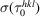 in the Laplace space (Klaus & Genzel, 2019 ▸.^2^

**Figure 13:**
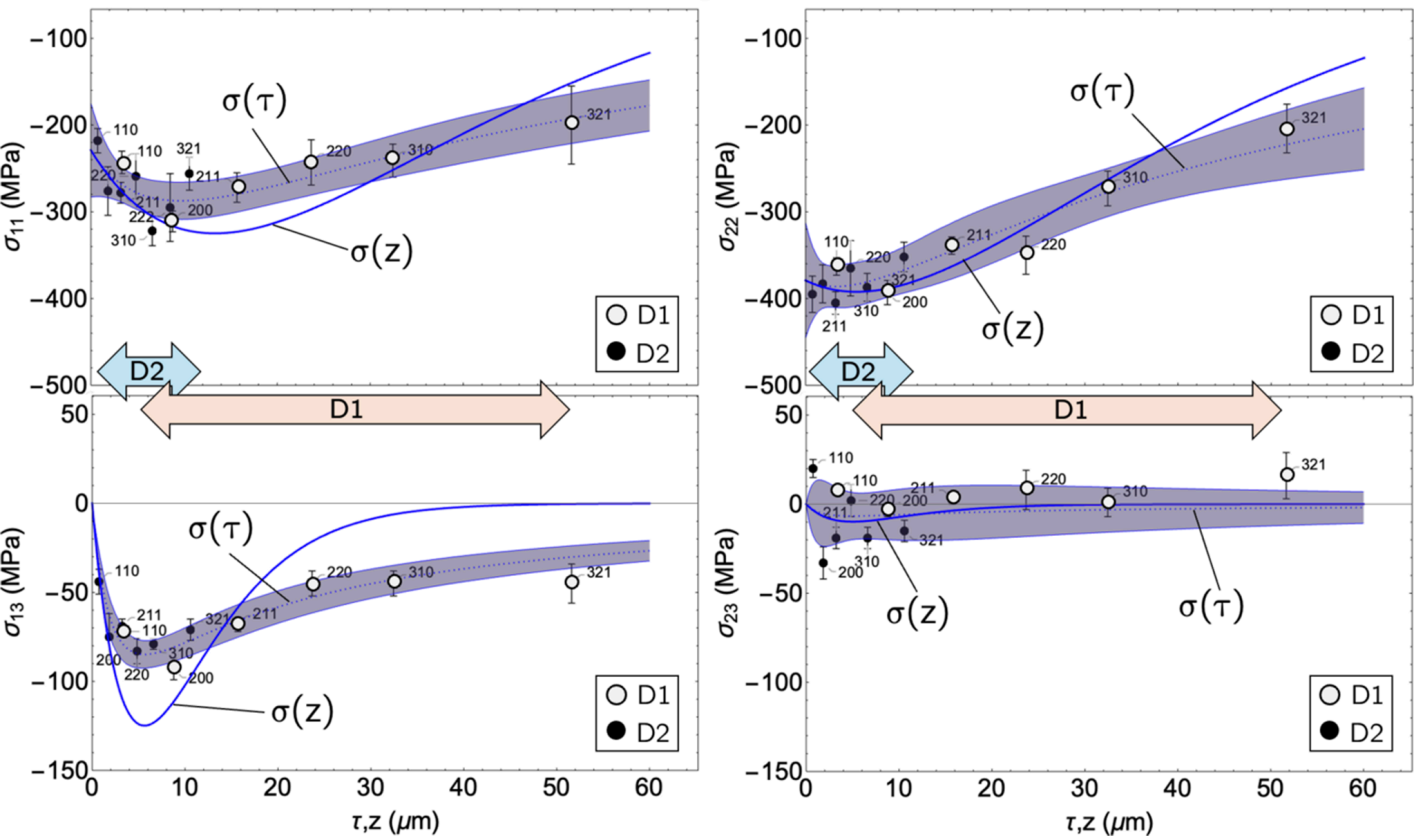
Near-surface residual stress depth profiles in real (*z*) and Laplace (τ) space of the analyzed specimen. The shaded areas mark the confidence intervals in the Laplace space, which comprise 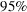 of the discrete data points. The double arrows denote the information depths covered by the two detectors D1 and D2.

In the second step, use was made of the transformation 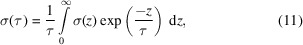
which relates the experimentally accessible stresses in the Laplace space to the stresses in the real or *z* space. The following approaches were used to describe the residual stress depth profiles in the real space: 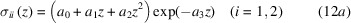
and 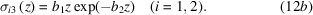
Approach (12*b*[Disp-formula fd12]) takes into account the boundary conditions that the out-of-plane stress components must fulfill at the free surface 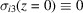. The Laplace transforms of the above expressions are obtained applying equation (11): 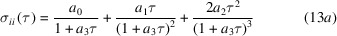
and 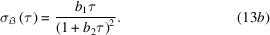
The unknown parameters were determined by least-squares fitting the equations (13*a*[Disp-formula fd21]) and (13*b*[Disp-formula fd22]) to the experimentally obtained discrete depth profiles 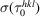. The results compiled in Fig. 13 ▸ clearly indicate the impact of the unidirectional mechanical surface treatment on the near-surface residual stress state.

It generates in-plane compressive stresses in both the grinding and transverse directions, with a higher level being observed in the latter. All stress components (with the exception of the shear stress component 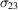 in the transverse direction, which is technically zero) occur in the form of more or less steep gradients and decrease with increasing depth. In particular, the stress components in the grinding direction, 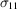 and 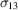, feature maxima below the surface, which are more pronounced in real space than in Laplace space. However, the shear stresses prove to be significantly less long range than the in-plane normal stresses, which remain within the covered information depth in the compressive state.

## 5. Discussion

A significant disadvantage of ED diffraction with laboratory X-ray sources is that the white *Bremsstrahlung* used for the measurements features a photon flux that is several orders of magnitude lower compared with that of the characteristic radiation. However, this disadvantage can be at least partially compensated for by the fact that complete diffraction spectra can be recorded simultaneously in various freely selectable but fixed measurement directions using two (as in the present work) or even more detectors. Concerning depth-resolved ED residual stress analysis, different measurement directions 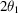 and 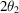 mean different average depth ranges, which are accessed during a χ scan. In this way, the number of discrete data points 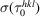 in the residual stress depth profiles can be doubled. At the same time, the information depth is extended in both directions: *i.e.* closer to the near-surface zone, which (in many cases) exhibits a strongly inhomogeneous residual stress state, and to deeper material regions, in which the residual stresses generated by mechanical surface treatment decrease or even change their sign.

The operation of both detectors in the same (here, horizontal) scattering plane facilitates the alignment of the diffractometer system considerably. So it eliminates the need to readjust (rotate) the Soller slit in front of an out-of-plane detector to limit the divergence in its scattering plane, which is necessary whenever the 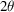 angle is changed (Apel *et al.*, 2018*b* ▸). However, on the other hand, as shown in Sections 2.2 and 2.3, the evaluation of the data obtained with the detector positioned in asymmetric diffraction geometry requires the introduction of correction terms in the fundamental equation of XSA. Stresses calculated from the slope of the regression lines fitted to the 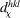
–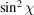 distributions must be multiplied by a factor of 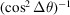. Even for large distances between the two detectors (in the present case, 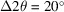), the deviation from the actual stress value is only 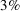 and should therefore be within the range of experimental uncertainties.

Both the detection of out-of-plane near-surface shear stresses and their separation from the in-plane normal stress components is based on the analysis of χ splitting generated by the shear components. For measurements in the symmetrical Ψ mode, data evaluation is straightforward, as complete/no splitting is observed in the grinding/transverse direction (see left column of Fig. 11 ▸). Therefore, it does not matter whether the two different χ branches were recorded by a single χ scan in the range 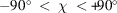 or by two χ scans in the range 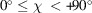 in opposite azimuths. However, for the asymmetric Ψ mode, represented in this paper by detector D2, the situation is different. Due to the inclination 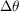 of the scattering vector 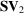 against the surface normal 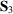 (for 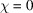), D2 also ‘recognizes’ the shear stress component in the transverse direction. Since 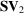 is always on the same side with respect to the χ plane of the Eulerian cradle, the partial splitting is visible in both grinding and transverse directions and therefore allows the separation of 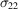 and 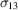 when χ scans are performed in the range 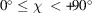 in the azimuths 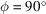 and 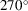 (*cf*. the lower diagram in the right-hand column of Fig. 11 ▸, and Fig. 12 ▸).

**Figure 14:**
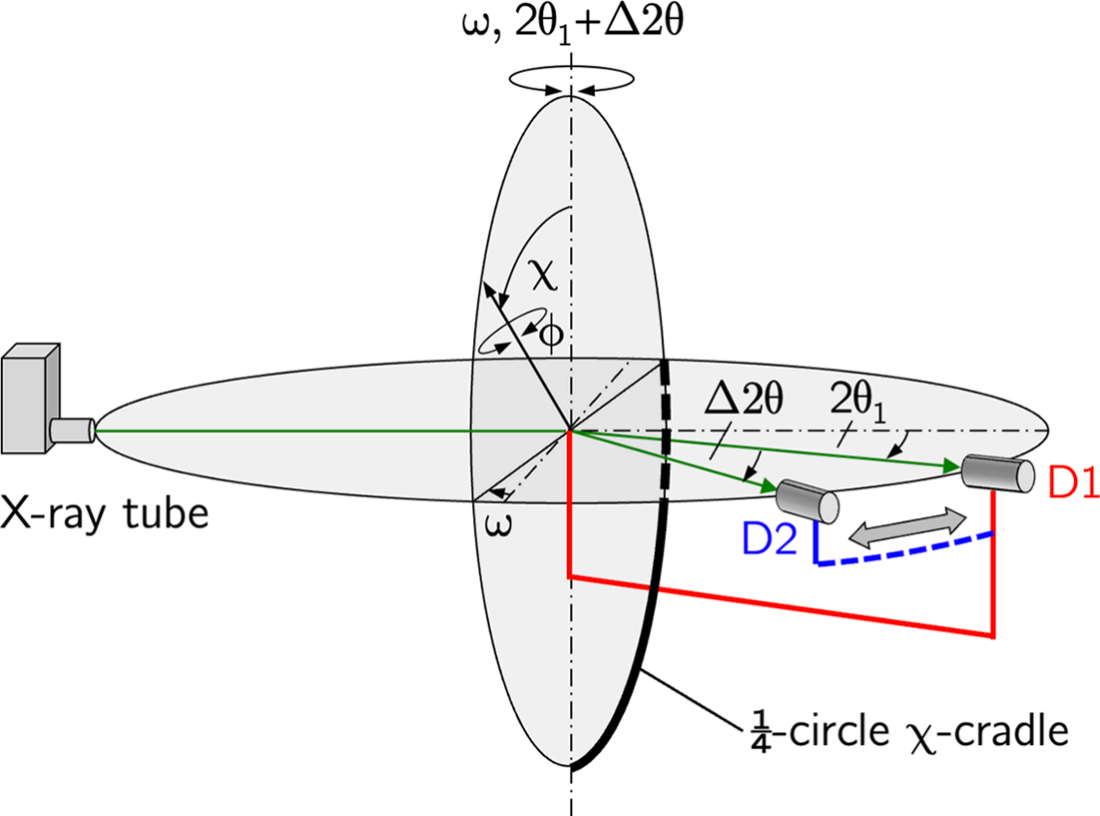
Sketch of a minimal instrumentation that enables depth-resolved ED-XSA with an equatorial two-detector setup.

The measurements to demonstrate the formalism introduced in this work were carried out using an eight-circle diffractometer. In principle, however, it seems conceivable to perform such analyses using a setup with a significantly reduced number of rotation/tilt axes. A possible minimum solution is depicted in Fig. 14 ▸. Assuming that detector D2 is mounted rigidly or mechanically movable on the arm for detector D1, this setup can be considered as a pseudo-four-circle diffractometer with a 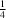-circle χ cradle and integrated ϕ-rotation table.

Finally, the proposed method is not restricted to the use of two detectors. Any additional detector aligned in the common scattering plane would cover another average depth range and therefore increase the number of nodes in the discrete Laplace stress depth profiles.

## Data Availability

The data presented in this study are available on request from the corresponding authors.

## Footnotes

1 We neglect the component  here for two reasons: (1) due to the boundary and equilibrium conditions valid for this component,  and , respectively, its impact can be assumed to be small within the depth range covered in the measurement; and (2)  has no influence on the relationships derived in the following for stress analysis under either symmetrical or asymmetrical diffraction conditions.

2 The derivation of  as the actual information depth for the generation of stress depth profiles in the Laplace space applies to symmetrical diffraction geometry (detector D1). However, since  [see equation (3*c*[Disp-formula fd5])] is close to zero, the assumptions underlying the derivation by Klaus & Genzel (2019 ▸) are also approximately fulfilled for detector D2.
